# Direct competition and potential displacement involving managed *Trogoderma* stored product pests

**DOI:** 10.1038/s41598-023-30618-8

**Published:** 2023-03-04

**Authors:** Michael J. Domingue, Yunke Wu, Kendra A. Vieira, Alana R. McGraw, Mandy Furtado, Christos G. Athanassiou, William R. Morrison, Scott W. Myers

**Affiliations:** 1 United States Department of Agriculture, Animal and Plant Health Inspection Services, Plant Protection and Quarantine, Science and Technology, 1398 West Truck Road, Buzzards Bay, MA 02542 USA; 2 Department of Entomology, Kansas State University, Manhattan, KS 66502 USA; 3 Department of Ecology and Evolutionary Biology, Cornell University, Ithaca, NY 14853 USA; 4 Laboratory of Entomology and Agricultural Zoology, Department of Agriculture, Crop Production and Rural Environment, University of Thessaly, Phytokou Str., 38446 Magnesia, N. Ionia Greece; 5 Department of Agriculture, Agricultural Research Service, Center for Grain and Animal Health Research, 1515 College Ave., Manhattan, KS 66502 USA

**Keywords:** Agroecology, Ecology, Ecology, Agroecology, Invasive species

## Abstract

The establishment of an exotic pest may require displacing local species with a similar niche. The potential of *Trogoderma granarium* to displace *Trogoderma inclusum* was explored in a stored product setting. We performed direct competition experiments varying commodity and temperature over different durations. At nine weeks *T. inclusum* outproduced *T. granarium* on all commodities at any temperature. However the proportion of *T. granarium* versus *T. inclusum* was greater at 32 °C compared to 25 °C. The nine-week production of *T. granarium* was best on wheat, while rice was optimal for *T. inclusum*. After 25 weeks, when adults were used at the start of competition, *T. inclusum* maintained an advantage in the direct competition. If larvae were used to initiate the competition for 25 weeks, the two species coexisted well at 25 °C, but *T. granarium* nearly excluded *T. inclusum* at 32 °C. Thus *T. inclusum* performs better in competition over shorter intervals when resources are plentiful, but *T. granarium* can be more successful over longer time periods, particularly when late instar larvae are involved. The finding suggests a real threat of introductions of *T. granarium* larvae to establish populations within grain storage infrastructure where *T. inclusum* is common.

## Introduction

*Trogoderma granarium* Everts (Coleoptera: Dermestidae), the khapra beetle, is a serious pest of most stored cereals and grains, as well as dozens of other commodities of plant and animal origin^1–4^. It has a native distribution in tropical and subtropical areas of the Indian subcontinent, and it has long been presumed that it develops well in warmer conditions. Its most long-lasting temporary introgression into the United States occurred in the warm arid southwestern portion of the country which has a climate most similar to its native range^5^. Significant infestations were later detected in warehouses in numerous other parts of the United States, requiring considerable mitigation efforts^6^. Other studies have documented how well the species performs at warmer temperatures^7–10^. Khapra beetle is considered a global threat to food security and has been listed as an A2 quarantine pest by the European and Mediterranean Plant Protection Organization (EPPO 2017)^11^. Because of this history of damage to the infrastructure of the global food production systems, many countries have developed strict regulatory regimes where this species is not endemic^12–14^.

*Trogoderma granarium* is also notable for the propensity of its larvae to enter facultative diapause when confronted by adverse environmental conditions, starvation or crowding^15,16^. This facultative diapause may confer an advantage for dissemination of the pest by human-assisted means because diapause will be broken when optimal temperature conditions coincide with the introduction of new food sources^15^. T*. granarium* larvae can maintain the diapause state for months to years before foraging again^17^. In the diapause state larvae can hide in cracks and crevices in food production, storage, and shipping facilities, and become difficult to detect and eliminate^11,18^. While these biological aspects of *T. granarium* are well-described, it is not known whether this behavior provides the species with an ability to compete with already established stored products pests.

*Trogoderma inclusum* LeConte, the larger cabinet beetle, is another dermestid beetle, which often infests stored products in the United States^19–22^. It also has a broad host range, with larvae that can be persistent^20^. It is uncertain whether the larger cabinet beetle originated in the US or in Europe, where it is also common^24,25^. Thus *T. inclusum* is a well established endemic pest in many areas where khapra beetle introduction would be a further concern. In addition to occupying a similar niche, it also utilizes a similar pheromone, with minor differences in composition of enantiomers^26,27^. It is not known to hybridize with other congeners such as *T. granarium*^19,28^. Thus, as a species that is an endemic pest of food storage facilities in the US, it is a potential competitor of *T. granarium*. It is of interest whether *T. granarium* could become established and outcompete *T. inclusum* under current or projected future environmental conditions^29^.

While risk management evalution would benefit greatly from determining the outcome of direct competition between closely related species such as *T. granarium* and *T. inclusum*, there are practical considerations hampering such research.. The larval stages of *Trogoderma* species are not distinguishable by any visible characteristics. Adults can be distinguished based upon the capacity for flight in *T*. *inclusum*^30^, which does not exist in *T. granarium*^31^, and other morphological characteristics that are apparent under sufficient magnification^32^. However, direct competition experiments will yield mixed populations where most individuals are in the larval stage. Furthermore, given the possibility of larvae remaining in diapause, relative adult and larval distributions may differ.

Molecular tools have the potential to assist in the proper identification of *Trogoderma* larvae in field or lab situations where morphologically similar species coexist. Currently available molecular tools include DNA barcoding and real-time PCR assays^33,34^. However, the former method is very costly (~ $10 per sample). Real-time PCR is more cost effective than sequencing (~ $1 per sample), but current assays only provide positive identification for *T. granarium* but not *T. inclusum*. A simple, efficient, and cost effective (~ $0.03 per sample) approach based on restriction fragment length polymorphism (RFLP) to rapidly distinguish between *T. granarium* and *T. inclusum* larvae was recently developed^35^. Because the two species exhibited characteristically distinct digestion patterns from the restriction enzyme NlaIII (New England Biolabs), which has no cut site for *T. granarium* but cuts the *T. inclusum* sequence into halves, the two species can be molecularly distinguished after agarose gel electrophoresis analysis.

Previous comparisons between *Trogoderma* species have only been indirect, performed in parallel under similar conditions, which has been done for the warehouse beetle, *Trogoderma variabile* Ballion and *T. granarium*^36^. However, there may be important interactions when these species directly compete, potentially including synergistic feeding on the resource, interference competition, chemical warfare, or consumption of dead individuals. In this study, we focus on measuring direct competition given various biotic and abiotic conditions. These conditions include temperature, commodity type, and whether the larval or adult stages initiate competition. Understanding direct competition will provide a better understanding of the conditions that might facillitate *T. granarium* displacing *T. inclusum*, which will help optimize risk management strategies.

## Results

### Larval size distribution of the species

**Figure 1:**
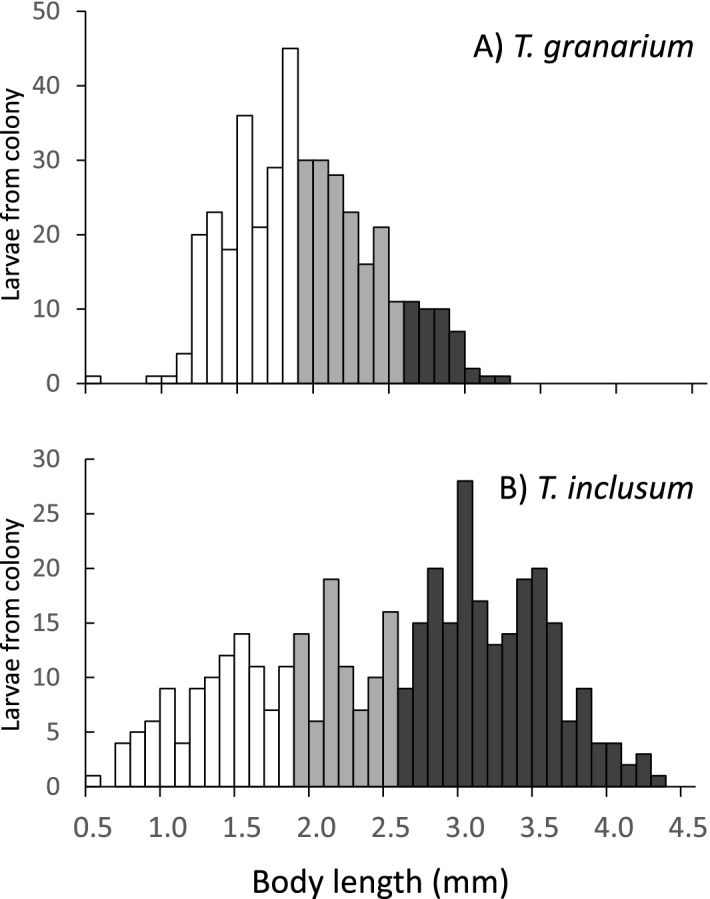
Size distribution of larvae sampled from lab colonies of *T. granarium* and *T. inclusum*. Size classes are indicated by the colors white (small), grey (medium), and black (large).

For larvae sampled from the colony jars, *T. granarium*, mean (± SE) length was 1.90 ± 0.02 mm, and for *T. inclusum* the mean (± SE) was 2.52 ± 0.04 mm. The means were significantly different using a 2-tailed t-test with the Satterthwaite approximation for unequal variances (*t* = 12.51, df = 605.95, *p* < 0.0001). The frequency distribution of larval measurements is shown in Fig. 1. After observing these data, the criterion for small (≤ 1.8 mm), medium (1.9–2.5 mm), and large (≥ 2.6 mm) were established. Pooling the data for both species, the distribution into these categories is 38, 32, and 31% respectively. Considering only *T. granarium*, the breakdown was 50% small, 40% medium, and 11% large, while the distribution for *T. inclusum* was 26% small, 21% medium, and 54% large.

### Nine week competition study

**Figure 2:**
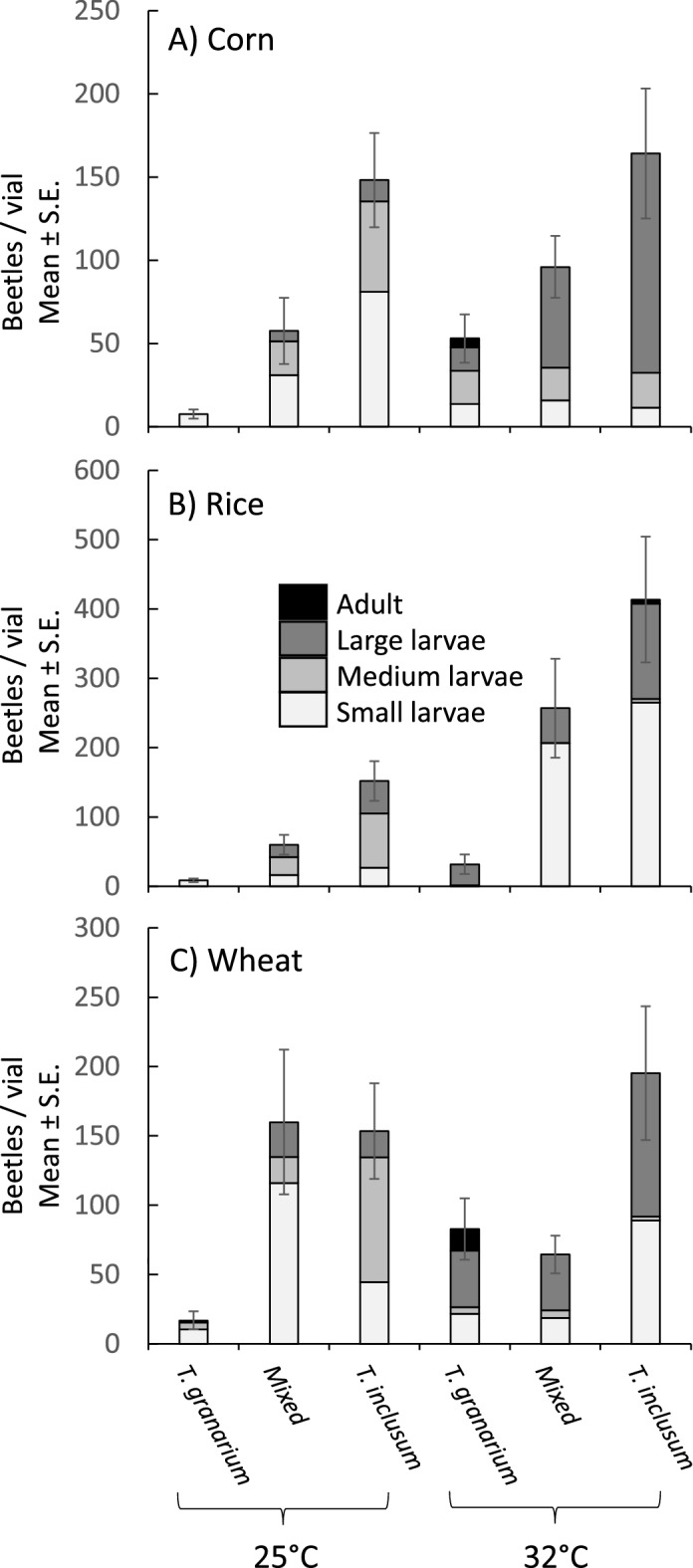
For the nine-week competition experiment, progeny production by *T. granarium*, *T. inclusum*, or mixed colonies at differing temperatures on each commodity type. Error bars apply to the entire population within each treatment including all larvae and adults.

In the nine-week study, many larvae were recovered in each type of treatment (Fig. 2). Only one pupa was identified (25 °C, mixed treatment wheat). There were a handful of adults in the parent treatments (*T. granarium* on corn and wheat, and *T. inclusum* on rice). When *T. inclusum* was reared at 32 °C, rice had the capacity to produce the most larvae. However rice also produced very low numbers of larvae in the treatments where only *T. granarium* was present. This pattern is also reflected in the significant interaction effects that commodity type had with the other factors (Table1). There was also a very strong main effect of species on progeny production in the nine-week study across all commodity types (Table 1), with a much greater number of *T. inclusum* when compared to *T. granarium*. Usually, the number of larave in the mixed treatments were at an intermediate level between the production observed in the two pure species.

**Table 1:** Analysis of variance for the effects of commodity type (corn, rice, or wheat), species (*T. granarium*, *T. inclusum*, or mix), and temperature (25 or 32 °C) on total progeny production, for the nine-week competition experiment (See Fig. 2, total df = 72)*.*

Effect	df	*F*	*P*
Commodity	2	4.95	0.0096
Species	2	33.25	< 0.0001
Temperature	1	13.17	0.0005
Comm*temp	2	7.87	0.0008
Comm*species	4	2.13	0.0864
Species*temp	2	1.44	0.2435
Comm*species*temp	4	3.23	0.0169

The effect of temperature was not as consistent. In corn there were no significant effects of temperature on progeny production (Fig. 2A), which can be confirmed by performing a separate analysis of variance using only the corn observations (df = 1, F = 2.73, *p* = 0.1117). However, the shading of the bars in Fig. 2A, indicates that larger larvae and adults were more common in the warmer treatments, indicating perhaps faster development, that had not yet resulted in a bigger population. For rice, warmer temperatures were associated with greater offspring production (Fig. 2A), which manifests in a significant effect of temperature in a separate analysis of variance for this commodity (df = 1, F = 15.92, p = 0.0005). In this case it is notable that many small larvae populate the warmer treatments with more offspring. Thus, for rice it appeared as if the warmer temperature stimulated development enough such that another generation of offspring had hatched, resulting in greater population totals. In wheat, the pattern observed in the mixed treatments was more complicated. The low temperature mixed treatments produced as much *T. granarium* progeny as *T. inclusum,* while the high temperature mixed treatments had progeny totals comparable with *T. granarium* alone (Fig. 2C). Unlike rice, but similar to corn, wheat had a greater proportion of large larvae and adults at the warmer temperature. This pattern again suggests faster development in the warmer conditions in wheat as was observed in the other commodities. However, in wheat and corn the apparent faster development in warmer temperatures did not accelerate development enough to start another generation of small larvae, as it had in rice.

**Figure 3:**
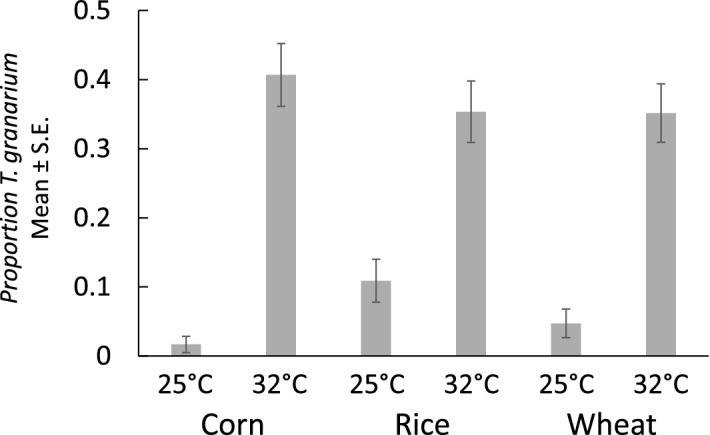
Proportion of *T. granarium* larvae (mean ± SE) in mixed nine-week treatments with *T. inclusum*, with respect to temperature and commodity type. The temperature effect is significant at *p* < 0.001 (see Table 1).

Of the 720 larvae sampled for molecular identification within the treatments treatments, we were able to positively identify 689 specimens (95.7%). A logistic regression was performed that considered the effect of commodity type, temperature, and length on the likelihood of a specimen being identified as *T. granarium.* Of those factors, only temperature was significant in affecting the proportion of *T. granarium* (Table 2). Figure 3 shows that at the higher temperature the proportion of *T. granarium* ranged from approximately 35% to 40% in the different commodities, while it was always less than 10% at the cooler temperature. Across all commodities, the mean (± SE) proportion of *T. granarium* at low temperature was 0.055 ± 0.013, and at high temperature was 0.370 ± 0.025. Despite the clear difference in length of the larvae in the parent colonies, the average lengths of the two species among the larvae identified within the competition treatments were nearly identical (*T. granarium*: 2.33 ± 0.06, *T. inclusum*: 2.21 ± 0.04). Thus, we presume that the proportion of *T. granarium* calculated in the sampled portion of the larvae offspring is representative of the entire population of mixed treatments, including the unsampled specimens.

**Table 2:** Logistic regression of the effects of commodity type, temperature and larvae size on the likelihood of a sampled larva being *T. granarium* versus *T. inclusum*, for the nine-week competition experiment.

Effect	df	Wald *Χ*^2^	*P*
Commodity	2	0.6583	0.7195
Temperature	1	75.3508	< 0.0001
Length	1	0.2553	0.6134

**Figure 4:**
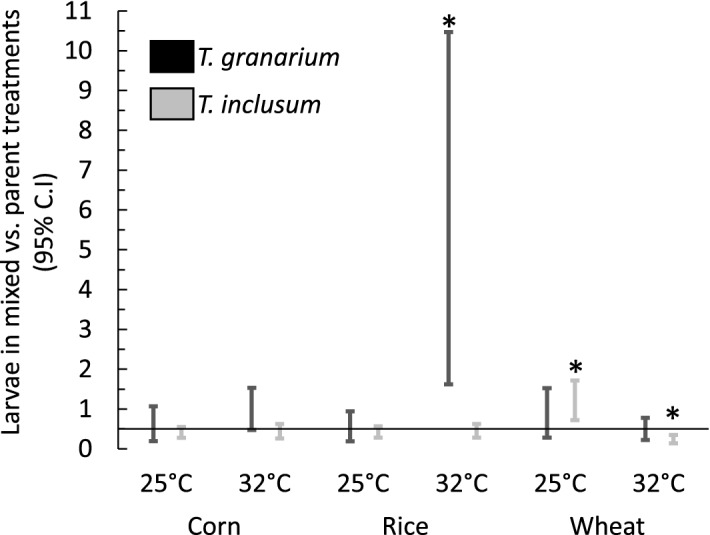
Comparison of the number of *T. granarium* and *T. inclusum* larvae in mixed treatments versus those in their parent populations at nine weeks. Estimates of this ratio were obtained by resampling of the populations given the probability of a larva being *T. granarium* at 25 °C (0.055 ± 0.013) or 32 °C (0.370 ± 0.025). *95% confidence intervals are presented in comparison to a value of 0.5, which is expected based upon the number of adults used to start the mixed and parental treatments.

Assuming that within each mixed treatment the probability of an larvae being *T. granarium* was 0.055 ± 0.013 at the lower temperature, and 0.370 ± 0.025 at the higher temperature, we created resampling events where we assigned species identity to each larva based upon comparing these events to the output of a random number generator. We simultaneously resampled the number of offspring in the parental treatments. If there were no significant competitive interactions in the mixed treatments, we would expect 50% of the number of both *T. granarium* and *T. inclusum* to occur in the mixed treatments, versus their number in the parental treatments. We repeated the resampling events 10,000 times to assess variation in these ratios. Figure 4 demonstrates that only in three instances does a 95% confidence interval based upon this resampling exclude a ratio of 0.5. These cases include *T. inclusum* on wheat, where there were more larvae than expected at 25 °C, but fewer than expected at 32 °C. Also, for *T. granarium* in warm conditions on rice, the 95% confidence intervals showed very high ratios. All values of the ratio within the interval were above 1.0, which indicates that when in competition with *T. inclusum* on rice, the average number of larvae produced was greater than when alone, although only half as many adults existed in the mixed treatments to initiate offspring production.

### Twenty-five week competition study

**Figure 5:**
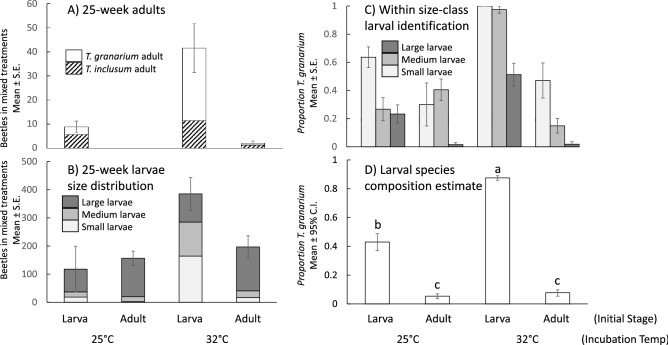
The populations of *T. granarium* and *T. inclusum* in mixed twenty-five-week treatments including the raw numbers of morphologically identified adults (**A**), small, medium, and large larvae (**B**), the proportions of *T. granarium* in molecularly identified larvae by size class (**C**), and resampled size-based estimates of the proportions of *T. granarium* among all larvae (**D**, Different letters indicate significant differences based on 95% confidence interval overlap).

After 25 weeks, the treatments started with larvae and held at 32 °C produced the most adult offspring at approximately 40 per treatment (Fig. 5A), of which 73% were *T. granarium.* The treatments started with larvae at 25 °C produced only about 9 adults per treatment (36% *T. granarium*) and those started with adults at 32 °C produced about two adults per treatment (40% *T. granarium*). There were no adults found in treatments initiated by adults at 25 °C. No pupae were found in the 25-week treatments, with most of the offspring observed being larvae (Fig. 5B). The pattern of larval production was similar to that of adults, with the greatest numbers observed when larvae were used to initiate the 32 °C treatments. Table 3 shows that the initial stage used for the treatments, the temperature they were stored at, and the interaction of these two factors are highly significant in impacting total progeny production (larvae and adults in Fig. 5 A-B combined). A Tukey-adjusted comparison of the total offspring production across treatments shows that the treatments started by larvae that were held at 32 °C were significantly more productive than the other three treatments, which did not differ from each other.

**Table 3:** Analysis of variance for the effects of the stage of insects used at the beginning (adult versus larvae) and temperature (25 or 32 °C) on total progeny production, for the twenty-five week competition experiment (See Fig. 5a,b, total df = 16)*.*

Effect	df	*F*	*P*
Beginning stage	1	9.19	0.0079
Temperature	1	3.06	0.0992
Stage*temp	1	5.22	0.0363

Of the 480 larvae molecularly sampled in the 25-week experiment, 474 (99%) were positively identified as *T. granarium* or *T. inclusum*. Figure 5C shows that the percentage identified to be *T. granarium* varied widely over the four treatments, and by size-class sampled. This percentage ranged from close to zero in large larvae when adults had been used to start the treatments, to 100% among small larvae when larvae had started the treatments at 32 °C. Logistic regression showed that the stage used to initiate treatments, temperature, the interaction of initiating stage and temperature, and larval length all had significant effects on the probability of any larvae in these mixed treatments being identified as *T. granarium* (Table 4). Among the larvae sampled in the four 25-week treatments, the mean (± SE) length was 2.08 ± 0.06 for *T. granarium* and 3.01 ± 0.11 for *T. inclusum*.

**Table 4:** Logistic regression of the effects of the stage of insects used at the beginning (adult versus larvae), temperature of incubation, the interaction of beginning stage with temperature, and larvae size on the likelihood a sampled larva is *T. granarium* versus *T. inclusum*, for the twenty-five week competition experiment*.*

Effect	df	Wald *Χ*^2^	*P*
Beginning stage	1	64.7195	< 0.0001
Temperature	1	19.6296	< 0.0001
Stage*temp	1	41.2113	< 0.0001
Size	1	67.6505	< 0.0001

Finally, when the larval distributions were estimated by repeated resampling of the larvae within each treatment, according to the size-based probabilities that each larva is *T. granarium* or *T. inclusum*, it became clear what the outcome of the competition was in each case (Fig. 5D). *Trogoderma granarium* was not able to establish itself well in either of the treatments that were initiated with adults, regardless of the temperature, representing less than 10% of the total larvae. However, substantial proportions of small larvae were still identified as *T. granarium* in these treatments (Fig. 2B), meaning that future success in competition was still possible. The share of *T. granarium* increased significantly to approximately 40% when larvae were used to start the 25 °C treatments, and then further to almost 90% when larvae were used for making the treatments stored at 32 °C.

### DNA cross-contamination experiment

It was noted in the competition experiment that the developing larvae were likely feeding on the dead bodies of adults because there were many treatments from which few intact adults were recovered, while pieces of adult wings or other cuticle pieces were readily apparent. The contamination control experiment included analyses of 20 T*. granarium* larvae feeding on *T. inclusum* larvae, 18 T*. granarium* larvae feeding on *T. inclusum* adults, 24 T*. inclusum* larvae feeding on *T. granarium* larvae, and 24 T*. inclusum* larvae feed on *T. granarium* adults. All specimens produced only the restriction length fragments characteristic of the live insects that had been fed dead specimens of the opposite species, suggesting no contamination when the two species were mixed.

## Discussion

The outcome of competition between *T. granarium* and *T. inclusum* was heavily influenced by both temperature and the initial life stage.. At 25 °C for the 9-week experiment, *T. inclusum* strongly outcompeted *T. granarium* on all commodities, confirming the general assumption that *T. granarium* is more adapted to warmer climates and will not thrive at cooler temperatures^10,33,37^. A similarly low percentage of *T. granarium* persisted on wheat after 25 weeks if adults were used to initiate the treatments at 25 °C. However, if larvae were used to start the treatments at this lower temperature, the two species faired similarly in the long-term. The improved outcome for *T. granarium* when larvae were used may be related to their ability to enter diapause under unfavorable conditions^15^, such as those encountered during intense competition. Females that have first experienced diapause as larvae lay more eggs as adults^38^, which might also explain the competitive ability of *T. granarium* larvae. Thus, assuming that many of the larvae taken from the crowded parental colonies were in diapause, they had a competitive advantage in the new treatments in competition with *T. inclusum*. The importance of the life stage initiating the treatments was further confirmed at 32 °C. At this warmer temperature, by 25 weeks, nearly all of the larvae were *T. granarium* when the larval stage was used to start the treatments.

However, when adults were used to initiate the treatments, the percentage of *T. granarium* was much smaller, even at the more favorable higher temperature, at almost 40% at 9 weeks, decreasing to under 10% at 25 weeks. Because adult *T. granarium* are shorter lived than *T. inclusum*, they may be at a severe disadvantage when transferred to new media at that stage. If only a few eggs were laid by one or two of the females that entered into the treatments, there may have also been a genetic bottleneck that impacted future fecundity, a process which has been documented in the red flour beetle, *Tribolium castaneum* (Herbst) (Coleoptera: Tenebrionidae)^39^. The weak competitive ability of adults, coupled with the known tendency of *T. granarium* to persist as larvae in diapause, underscores the importance of detecting the presence of larvae in *T. granarium* management plans. Similarly, a competition experiment using larvae of *T. granarium*, and adults of the rice weevil, *Sitophilus oryzae* (L.) (Coleoptera: Curculionidae) and the lesser grain borer, *Rhyzopertha dominica* (F.) (Coleoptera: Bostrychidae) and found that *T. granarium* could rapidly outcompete the other two species at temperatures above 30 °C^9^.

The differences among the commodity types seemed to involve developmental rates, which were more apparant for *T. inclusum*. Other studies have also shown effects of commodity types on *T. granarium*^37^ and other *Trogoderma* species^30^. At 25 °C, none of the treatments seemed to progress to a second generation within nine weeks (Fig. 3), but there were greater proportions of medium to larger *T. inclusum* larvae on wheat and rice compared to corn, suggesting faster development. At 32 °C, more noticeable effects on development rate occurred. For *T. inclusum*, there was a second generation in rice, and a clearer indication that development on wheat was faster than on corn. However, development was best for *T. granarium* on wheat, followed by corn and then rice. Subtle interactions occurred between the species under direct competition. *Trogoderma granarium* performed significantly better than expected in the mixed treatments at higher temperature on rice (Fig. 5) The impact of this finding may warrant further examination given that there is also evidence of differences in *T. granarium* performance on different rice cultivars^40,41^. At the same time, *T. inclusum* was significantly affected by the presence of *T. granarium* on wheat, with a positive effect at 25 °C, but a negative effect at 32 °C. As is the case with rice, it may also be important to further explore this interaction for understanding the risk of introduction of *T. granarium* to wheat stores already infested by *T. inclusum.* Also, further complicating such an interaction might be different performance on varieties of wheat^44^.

It was not surprising that the larvae taken from the parental colonies of the two species were of different lengths on average. It has generally been informally observed that the adult *T. inclusum* in these lab colonies are larger than the *T. granarium* adults. Furthermore, Fig. 1 indicates a broader distribution for *T. inclusum,* with multiple peaks, indicating the progression through instars. However, the size distribution in *T. granarium* is normally distributed with a maximum frequency just below 2 mm, likely indicative of a tendency toward diapause at this length. When performing the treatments at 9 weeks, before competition strengthens, there was no evidence of any differences in sizes between the species. However, after 25 weeks, size differences between the two species reemerged, suggesting that competition may have triggered diapause in *T. granarium*. Increased larval densities are among the critical factors that induce diapause in *T. granarium*^43^.

Overall, the results support the prospect that *T. granarium* can assert competitive strength, and displace established populations of *T. inclusum*. In particular, warmer temperatures and initial presence of larvae favor this outcome. Current models that evaluate likelihood of *T. granarium* establishment in quarantine areas tend to focus on risk based upon climactic data and interception patterns^44^, but do not consider the potential interactions with native *Trogoderma* in risk assessment. While other studies have suggested that *T. granarium* may be quite adept at displacing grain pests from other taxa^9^, the larger picture of the role of biotic interactions in risk assessment and mitigation remains unclear. The current study shows that local populations of *T. inclusum* may sometimes provide protection from *T. granarium*.

## Methods

### Insects and rearing conditions

All experiments were conducted at the United States Department of Agriculture Animal and Plant Health Inspection Service (USDA-APHIS), Science and Technology, Forest Pest Methods Laboratory, Buzzards Bay, MA, USA. *Trogoderma granarium* and *T. inclusum* colonies were reared in ~ 1 L glass jars in separate rooms at 30 °C, 60% relative humidity and continual darkness in chambers as described elsewhere^45^. *Trogoderma variabile* larvae were derived from a field strain collected from eastern Kansas. The *Trogoderma granarium* culture derives from a strain collected in Pakistan.

### Larval size in lab colonies

Four hundred larvae from each of the parent colonies were sampled from paper strips placed above the diet. Larvae were then killed by being frozen at −40 °C for 48 h. The length of each larva was then measured to the nearest tenth of a mm using a microscope fitted with an ocular micrometer (Heerburg M3, Wild, Switzerland). The pooled distribution of larval sizes of the two species was assessed to categorize larvae as small, medium, or large in subsequent experiments, with cutoff points that placed approximately one-third of the larvae into each of these categories. Experimental treatments were always performed in an environmental chamber within an additional separate room away from the permanent colonies, with 60% relative humidity and continual darkness, set to 25 °C or 32 °C.

### Nine-week competition study

The first experiment assessed competition between *T. granarium* and *T. inclusum* over nine weeks at two different temperatures in three different commodities. The experiment had a 3 × 2 × 3 factorial design, i.e. for commodity, temperature and species identity, with each treatment combination being replicated five times. The commodities selected were common agricultural products, wheat, corn, and rice obtained from local farmers in Kansas as described elsewhere^46^. The commodities were stored in ventilated screw-top plastic containers in the laboratory at ambient conditions for approximately one month before initiating the experiment. The two temperatures used were 25 °C or 32 °C, which were set in different Percival environmental chambers within the same room. There were three levels of species identity, i.e. pure *T. granarium*, pure *T. inclusum*, or a mixture of the two. In each case, 15 g of the material was placed in a jar with a screw top lid with a 2 cm diameter screened cutout in the lid for ventilation. Eight males and eight females were placed in each of these vials and placed in an environmental chamber for the duration of the nine-week period. At the end of this period, each jar was placed in a −40 °C freezer within sealed ziploc freezer bags for a minimum of 48 h to kill all of the beetles and allow them to be counted and sampled for length and molecular identity. The first treatment was to use 8 male and 8 female adult *T. granarium* in each jar, while the second was to similarly use only *T. inclusum*. The third species treatment was to directly measure competition by mixing the two species by including 4 males and 4 females of both species in a jar together. Approximately two weeks were needed to collect enough adults from the source colonies to perform the experiment. The treatments were initiated as soon as the adults were collected, with each of the 18 treatments within a replicate completed before moving on to the next replicate.

We established the goal of collecting 24 larvae from each replicate of the six different treatments of mixed *T. granarium* and *T. inclusum* for molecular identification. The length of each larva selected was measured using the ocular micrometer. If enough larvae were available, we also attempted to divide these collections such that 8 small, 8 medium, and 8 large larvae were selected. However, if there were not enough larvae in one size class, we sampled from the remaining size classes equally until this quota was met. In a handful of the treatments, 24 total larvae were not available within an entire jar. In these cases, we took some additional samples from other similar treatments that had greater progeny numbers to fully use the resources set aside for the experiment. The remaining larvae and adults also counted and life stages were recorded. Very few adults were collected from mixed species treatments, all of which appeared to have been dead and older at the time of collection, and likely to have been the parents used to initiate the treatments. Thus, no attempts were made to identify these adults to species level. All the larvae not selected were sorted as small, medium, or large when they were counted, but their exact lengths were not recorded. The large volume of larvae in some treatments made such measurements impractical, while many larvae could easily be visually grouped as large or small without pausing for a precise measurement. Larvae from the mixed treatments were identified by the aforementioned RFLP method.

### Twenty-five-week competition study

We repeated the procedures described above for the nine-week experiment for twenty-five weeks with some modifications. For this longer-term experiment wheat was the only commodity used, because we had learned that the commodity type had not caused an extremely strong effect in the shorter study. Only mixed-species treatments were performed in this instance, with no single-species controls. By eliminating these treatments, in this experiment, we were able to add a treatment in which the beetles used to start the colonies were large larvae that were likely to be later instars from the parent colonies rather than adults. However, we did not select from the very largest larvae, which are more likely to be female to avoid biases. We again used 8 *T. granarium* and 8 *T. inclusum* individuals to start each treatment. When adults were used, a 50% sex ratio was again assigned to each treatment, but it was not possible to determine the sex of larvae. As we had done with the 9-week study we duplicated this experiment at 25 °C and 32 °C. Thus, this experiment had a 2 × 2 factorial design with temperature and initial life stage being the two factors. Each of these treatments were again replicated five times. At the end of the 20-week period, we identified 24 larvae per replicate using the RFLP method, recording the exact length of each specimen to 0.1 mm, again attempting to select 8 small, 8 medium, and 8 large larvae if available. We also counted all remaining larvae, categorizing each as small, medium, or large. A much greater number of adults were found in many of these treatments in comparison to the nine-week treatments. All adults were collected and identified to species and sex as described by Gorham^32^.

### DNA cross-contamination experiment

In mixed treatments the two species make physical contact and may even feed on dead bodies of the other species, therefore it is critical to evaluate whether the RFLP method was prone to sample contamination. We performed additional experiments to examine whether such contact and feeding might contaminate the DNA extracted from larvae.

For this experiment, 50 small to medium sized larvae of *T. granarium* or *T. inclusum* were placed in screw-top jars with 15 g of wheat. They were then fed with 50 dead larvae or adults of the opposite species, which had been killed by freezing. The feeding was repeated in two successive weeks. The experiment was terminated four days after the final feeding and 18 d after the experiment initiation. At the end of the experiment live larvae were removed from the vials and placed in 95% ethanol for molecular identification. For each of the four treatment combinations (*T. granarium* larvae feed on *T. inclusum* larvae, *T. granarium* larvae feed on *T. inclusum* adults, *T. inclusum* larvae feed on *T. granarium* larvae, *T. inclusum* larvae feed on *T. granarium* adults), there were two replicate jars. From within each replicate, 6 to 12 larvae were identified by the RFLP method. The presence of fragments from both species would indicate likely contamination of the samples via the gut contents containing DNA of the dead larvaecprovided, or the exoskeleton being directly exposed to the dead insects or feces containing remnants of consumed cadavers.

### Statistical analyses

When applicable, analysis of variance or logistic regression was performed using Proc GLM and Proc Logistic in SAS v.9.4 (Carey, NC). For analyses of variance using Proc GLM, the LSMEANS option was used for individual comparisons, using a Tukey adjustment. Resampling using the random number generator in Excel was also used in a couple of specific contexts to create distributions of means of the populations of each species within the mixed treatments, based upon the identification of the subsamples of larvae using molecular techniques. In any such cases the resampling events were repeated 10,000 times. The resampled means were used to create 95% confidence intervals for hypothesis testing.

There were no indications of size differences between the larvae in the nine-week population, meaning that the population of each species could simply be extrapolated from the total number of larvae counted and those identified. A more complicated procedure was needed to estimate the number of larvae in the four mixed treatments of the 25-week experiment, because differences in the species composition between small, medium, and large larvae emerged in the 25-week experiment. This difference required adjusting the statistical model for estimating larval population composition. Thus, for the latter experiment, a procedure was envoked to resample larvae within each size category based upon the subsample of molecularly identified specimens within each size class. This allowed us to adjust our estimate of the ratio of the two species in each treatment using the subsample of molecularly identified larvae in combination with the raw numbers.

Resampling was also invoked to compare the parental populations to the mixed treatments in the 9-week study. Using the estimated proportion of *T. granarium* vs. *T. inclusum* in each treatment, we resampled all larvae in the mixed treatments, assigning them to either species using a random generator. At the same time, we also resampled the population sizes of the parent populations with replacement. We then compared the ratio of the average estimated population in the mixed treatments to the population of the parent treatments for each resampling event. Because the mixed treatments had half the number of each species compared to the parental treatments, a ratio of 0.5 would be expected. Higher or lower numbers would indicate a competitive advantage or disadvantage of that species when encountering the other species.

### Ethical statement regarding animal and plant experimentation

For the plant and animal material, all methods included in this study are in accordance with relevant guidelines. Additional institutional guidelines for use of quarantine insects were followed. The animal experimentation follows protocols outlined in accordance with the permit issued for the Forest Pest Methods Lab bt the USDA Animal and Plant Health Inspection Services Plant Protection and Quarantine for rearing khapra beetle on the described commodities (P526-20-03209). The results of the study are reported in accordance with ARRIVE guidelines (https://arriveguidelines.org) for animal experimentation.

## Data Availability

The data presented in this study are available from the corresponding author.
